# One-year evaluation of a new restorative glass ionomer cement for the restoration of non-carious cervical lesions in patients with systemic diseases: a randomized, clinical trial

**DOI:** 10.1590/1678-7757-2020-0311

**Published:** 2020-10-19

**Authors:** Fatma Dilsad OZ, Ece MERAL, Esra ERGİN, Sevil GURGAN

**Affiliations:** 1 Hacettepe University School of Dentistry Department of Restorative Dentistry Sihhiye Ankara Turkey Hacettepe University , School of Dentistry , Department of Restorative Dentistry , Sihhiye , Ankara , Turkey .

**Keywords:** Flowable composite resin, Non-carious cervical lesions, Resin composite

## Abstract

**Objective:**

This randomized and clinical trial aimed to evaluate the performance of a new restorative Glass Ionomer Cement (GIC) for the restoration of non-carious cervical lesions (NCCLs) of patients with systemic diseases compared with a posterior resin composite after 12 months.

**Methodology:**

134 restorations were placed at 30 patients presenting systemic diseases by a single clinician. NCCLs were allocated to two groups according to restorative system used: a conventional restorative GIC [Fuji Bulk (GC, Tokyo Japan) (FB)] and a posterior resin composite [G-ænial Posterior (GC, Tokyo Japan) (GP)] used with a universal adhesive using etch&rinse mode. All restorative procedures were conducted according to manufacturer’s instructions. Restorations were scored regarding retention, marginal discoloration, marginal adaptation, secondary caries, surface texture, and post-operative sensitivity using modified United States Public Health Service (USPHS) criteria after 1 week (baseline), 6, and 12 months. Descriptive statistics were performed using chi-square tests. Cochran Q and Mc Nemar’s tests were used to detect differences over time.

**Results:**

After 12 months, recall rate was 93% and the rates of cumulative retention failure for FB and GP were 4.9% and 1.6% respectively. Both groups presented similar alpha rates for marginal adaptation (FB 86.2%, GP 95.5%) and marginal discoloration (FB 93.8%, GP 97%) at 6-month recall, but FB restorations showed higher bravo scores than GP restorations for marginal adaptation and marginal discoloration after 12 months (p<0.05). Regarding surface texture, 2 FB restorations (3.1%) were scored as bravo after 6 months. All restorations were scored as alpha for secondary caries and postoperative sensitivity after 12 months.

**Conclusion:**

Although the posterior resin composite demonstrated clinically higher alpha scores than the conventional GIC for marginal adaptation and discoloration, both materials successfully restored NCCLs at patients with systematic disease after a year.

**Clinical relevance:**

Due to its acceptable clinical results, the tested conventional restorative GIC can be used for the restoration of NCCLs of patients with systemic diseases.

## Introduction

Non-carious cervical lesions (NCCLs) develop in exposed root surfaces due to several factors, including abrasion, friction, and stress forces, ^1^ and they are presented in different clinical forms, such as shallow grooves, large wedge-shaped defects with sharp line angles, and disc-shaped lesions. They are mostly observed on incisors, canines, and premolars, and they have been shown to affect the maxillary teeth more than the mandibular teeth. ^2^

Abrasion of the root is associated with esthetic problems, hypersensitivity, and bacterial plaque accumulation, which are considered the main reasons for treatment and restoration. ^3^ Multiple factors are involved in the occurrence of NCCLs, and the lesions depth and width can vary. ^4^ Although their etiology differs among cases, the prevalence of NCCLs is increasing with the aging of the population, and thus the risk of developing systemic disease is also increasing. ^5 - 7^

Either glass ionomer cement (GIC) or resin composite in combination with resin-based adhesive are preferred to restore NCCLs. The choice is usually based on the clinician’s preference and ease of handling the material without considering the durability of restorations supported by strong clinical evidence.

A number of clinical trials have assessed the performance of GICs for restoring NCCLs, and these trials have demonstrated acceptable clinical results. ^8 - 11^ Fluoride release from GICs may provide effective tooth surface protection against demineralization, also supporting teeth integrity. ^12^ The weaker physical properties of GICs are considered as disadvantages compared to resin composites, along with their poor esthetic properties (e.g., limited range of shades). ^1 , 12^ GICs bond to teeth by micromechanical and chemical bonding, and therefore they are considered self-adhesive materials. ^13^

Recently, a new conventional GIC system (Fuji Bulk; GC, Tokyo Japan) was introduced. ^14^ This new GIC offers a faster setting time, so that it can be used for older patients and patients who are unable to stay in the dental chair for a long time. ^14^ The manufacturer claims that a purpose-designed glass filler and a new higher-molecular-weight polyacrylic acid enable this GIC to have increased resistance. ^14^ Hence, this material may be a better choice for use in geriatric patients and individuals with dry mouth or active caries, and also for patients with systemic diseases who have to struggle with more challenging oral conditions than healthy individuals.

Increases in life expectancy have been followed by changes in the morbidities rates that mostly affect older individuals. Chronic and systemic diseases, such as heart disease, ^15^ cancer, ^16^ and diabetes mellitus, ^17^ are most likely to affect older individuals, and these diseases and the medicine used to treat them can affect the flow of saliva and dental health. Oral environment in such patients may not benefit from the normal buffering capacity of saliva. Therefore, restorative materials for these patients should be chosen carefully, and treatments may require more attention due to their sensitive condition. A bioactive, biocompatible, and fast-setting fluoride-releasing GIC restorative material with good resistance to wear and acidic conditions may be suitable for such patients, particularly for restoring lesions where the restorative material are in contact with vulnerable gingival tissues.

Note that the limited data reported to date regarding the performance of GICs on NCCLs have been obtained from studies carried out on healthy individuals with no systemic disease or gingival problems. ^18^ This is the major limitation to investigate clinical scenarios for most cases where NCCLs, systemic disease, and periodontal problems may occur with them. Therefore, this study evaluated the clinical performance of a new conventional GIC compared to a resin composite applied with a universal adhesive for the restoration of NCCLs in patients with systemic diseases. The null hypothesis was that there would be no differences in clinical performance between conventional GIC and resin composite restoration.

## Methodology

This was a randomized and controlled clinical trial. The study protocol was approved by the Institutional Research Ethics Committee for Clinical Investigations (KA-19010) and registered at ClinicalTrials.gov (NCT04266210). Patients provided written informed consent prior to the beginning of any treatment. The experimental design followed the Consolidated Standards of Reporting Trials (CONSORT) statement. ^19^

### Sample size calculation

The sample size was calculated using G*Power software (version 3.1) with 95% confidence interval, 90% power, and 0.40 effect size in the chi-square test. The highest degree of freedom was assumed to be 5 and the minimum restoration number was determined to be 51 per group. Considering the possibility of dropouts during the study period, the sample size was increased to 67 in each group and a total of 134 restorations were performed.

### Patient screening

**Figure 1:**
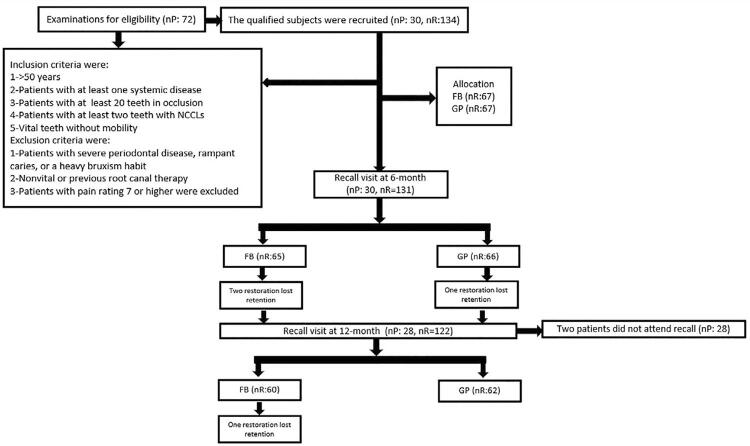
Flow diagram of the study. FB: Fuji Bulk, GP: G- ænial Posterior, nP: number of patients, nR: number of restorations

A clinician recruited participants who met the inclusion criteria ( Figure 1 ) among patients seeking routine dental care from the Restorative Dentistry Department. Non-retentive lesions with a cavosurface margin involving at most 50% of the enamel were included. Assessments were carried out using an explorer, a mouth mirror, and a periodontal probe. The cold test was also performed for sensitivity to avoid the inclusion of patients with severe hypersensitivity. Patients were asked to grade their pain on a scale ranging between 0 and 10 and some patients were excluded if the pain rating was 7 or higher.

Patients older than 50 years who had at least one systemic disease ( Table 1 ) were included in the study. All patients had at least 20 teeth ^20 , 21^ in occlusion and at least 2 teeth with NCCLs that had to be restored. Teeth had to be vital without mobility. Patients with severe caries or heavy bruxism were excluded. A total of 72 patients were screened for the study and 30 participants were selected. A total of 134 NCCLs were restored in 30 (17 female, 13 male) patients with a mean age of 61.8 years. The study design is presented in Figure 1 .

**Table 1:** Systemic diseases of patients included in the study

Systemic disease	Number of patients
Hypertension	16
Cancer patients in remision	6
Diabetus Mellitus	5
Hemophilia	1
Heart disease – coronery artery disease	4
Rheumatoid arthritis	2
Chronic obstructive pulmonary disease	2
Lupus eritematozus	1

### Randomization

Randomization was carried out by another clinician who was not involved in the research protocol. The teeth were randomized for each of the two restorative treatments by a random number table generated by the program “Research Randomized Program” (http://www.randomizer.org/form.htm). Similar numbers of restorations were placed in both groups, and each patient received at least two restorations. In some cases, more lesions were restored following the same randomization protocol.

### Restorative treatments

**Figure 2:**
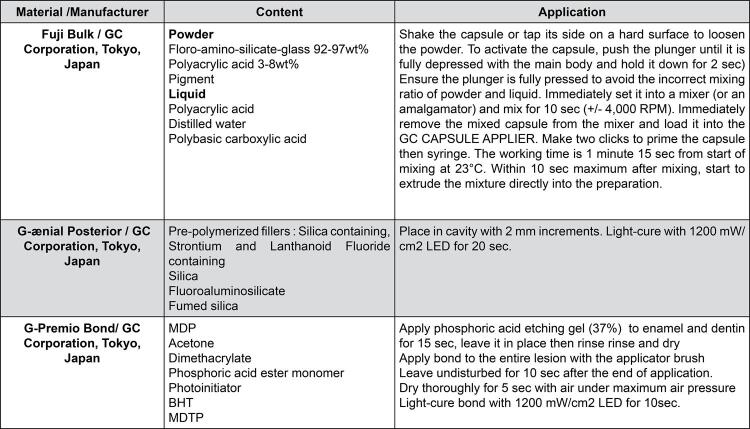
Application procedures of materials used in the study MDP: 10-Methacryloyloxydecyl dihydrogen phosphate, BHT: Butylated hydroxytoluen, MDTP: Methacryloyloxydecyl dihydrogen thiophosphate.

The materials used in the study are listed in Figure 2 . Patients with lesions 1~3 mm deep, were included in the study. Before starting the restorative procedures, the distribution of demographic characteristics of patients according to sex and age were recorded ( Table 2 ). Data on NCCLs according to tooth type, arch, degree of angle, cervicoincisal height, buccolingual depth, and restorative systems are presented in Table 3 .

**Table 2:** Demographic Characteristics of Patients

	Number of patients
Gender distribution	
Male	13
Female	17
Age distribution	
50-59	18
60-69	12

**Table 3:** Characteristics and Distribution of NCCLs

	Fuji Bulk	G-ænial Posterior
Tooth distribution		
Premolars	44	55
Molars	23	12
Shape/degree of angle		
<90	21	15
90-135	46	52
>135	0	0
Cervicoincisal height		
<1.5	0	0
1.5-2.5	29	22
>2.5	38	45
Buccolingual depth (mm)		
01/fev	16	9
02/01/2003	51	58
Arch distribution		
Maxillary	28	34
Mandibular	39	33

Patients received dental prophylaxis and oral hygiene instructions one week before treatments. The gingival index (GI) and plaque index (PI) of each tooth were determined before treatment. ^22^ In total, 13 (9.7%) teeth were scored as 0 (no bleeding), 69 teeth (51.5%) were scored as one (some bleeding after probing), 45 (33.6%) teeth were scored as two (bleeding immediately after probing), and seven (5.2%) teeth were scored as three (bleeding on probing spreading toward the marginal gingiva). The overall PI for patients was 0.96 (SD: 0.32, *n* =134). Patients’ salivary flow rate and saliva pH were checked before starting treatments. Mean unstimulated and stimulated salivary flow rates were 0.19±0.75 and 0.82±1.23 mL/min, respectively, both flows were low. Six patients had salivary pH 6.8–7.8 (healthy saliva) and 24 patients had moderately acidic saliva (pH 6.0–6.6).

The lesions were cleaned with a slurry of pumice and water on slow-rotating rubber cup in a slow-speed hand piece, rinsed, and dried. One operator performed all restorations; despite having more than 8 years of experience, the operator performed 10 restorations with each test material in patients not included in the study before starting the trial. Each restoration was scored as alpha by the two previously calibrated operator. At this point, the operator was considered calibrated to perform restorative procedures during the study. ^23^ Restorative procedures were carried out after isolating the lesions using cotton rolls. Restorative materials (Fuji Bulk [FB, *n* =67] and G-ænial Posterior [GP, *n* =67]) were applied according to the respective manufacturer’s instructions listed in Figure 2 . Resin composite restorations were light-cured using an LED light-curing unit (Radii Plus; SDI, Victoria, Australia) set at 1200 mW/cm ^2^ . The intensity was checked regularly using a radiometer (Benlioğlu radiometer; Benlioğlu Dental, Ankara, Turkey) before each use. All restorations were contoured using flame-shaped fine finishing diamond burs (Diatech, Charleston, SC, USA) in a high-speed hand piece under water spray, and then polished with Optidisc discs (Kerr Corporation, Orange, CA, USA). Coating material (EQUIA Forte Coat, GC, Tokyo, Japan) was applied with a microbrush to the surfaces of FB restorations and light-cured for 20 s.

### Blinding and calibration for clinical evaluation

Before starting the evaluations, two experienced examiners other than the operator were trained for both intra-examiner and inter-examiner reliability. For this purpose, they observed 10 photographs representing the scores for each criterion. The percentage of agreement between examiners was at least 85%. The examiners who were not involved with the procedures of restoration and blinded to the group assignment performed the clinical evaluations independently using mirrors, probes, and air streams. Disagreements were resolved by consensus during evaluations. Patients were also unaware and blinded to the treatments (i.e., which teeth received which type of restoration).

Restorations were evaluated at baseline (1 week after restoration placement) and 6 and 12 months after placement for retention, marginal adaptation, marginal discoloration, surface texture, and postoperative sensitivity according to modified United States Public Health Service (USPHS) criteria. ^24^ New and empty evaluation forms were filled by the examiners to remain blinded to group assignments at recall.

Restoration retention rates were calculated using an equation (Cumulative failure % = [(PF+NF)/(PF+RR)] x 100%, PF = number of previous failures before the current recall; NF=number of new failures during the current recall; RR=number of restorations recalled for the current recall). ^21 , 25^

### Statistical analyses

Cohen’s kappa statistic was used to test inter-examiner agreement. Comparisons of the groups in each category were performed using Pearson’s chi-square test. The baseline scores were compared to those at recall visits using Cochran’s Q-test followed by McNemar’s test. Kaplan–Meier analyses were performed to compare cumulative retention rates. All statistical analyses were carried out using IBM SPSS version 22.0 (SPSS, Chicago, IL, USA). In all analyses, *p* <0.05 was considered as statistical significance.

## Results

Clinical evaluation scores of restorations are shown in Table 4 . Most restored teeth were premolars (73%). After randomization, 53% were placed at the mandibular arch and 47% were placed at the maxillary arch. At baseline, all restorations scored alpha regarding the modified USPHS criteria evaluated (retention, marginal adaptation, marginal discoloration, surface texture, secondary caries, and postoperative sensitivity).

**Table 4:** Clinical evaluation outcomes of restorations

Evaluation Criteria	Score	Baseline n (%)		6-month n (%)		12-month n (%)	
		FB (67)	GP (67)	FB (67)	GP (67)	FB (60)	GP (62)
Retention	Alfa	67	67	65	66	59	62
		(100)	(100)	(97)	(98.5)	(98.3)	(100)
	Charlie			2	1	1	
				(3)	(1.5)	(1.7)	
Marginal Adaptation	Alfa	67	67	56	63	41	58
		(100)	(100)	(86.2)	(95.5)	(69.5)	(93.5)
	Bravo			9 ^s^	3	18 ^s^	4 ^s^
				(13.8)	(4.5)	(30.5)	(6.5)
	Charlie						
Marginal Discoloration	Alfa	67	67	61	64	47	58
		(100)	(100)	(93.8)	(97)	(79.7)	(93.5)
	Bravo			4 ^s^	2	12 ^s^	4 ^s^
				(6.2)	(3)	(20.3)	(6.5)
	Charlie						
Surface Texture	Alfa	67	67	63	66	52	59
		(100)	(100)	(96.9)	(100)	(88.1)	(95.2)
	Bravo			2		7	3
				(3.1)		(11.9)	(4.8)
	Charlie						
Postoperative Sensitivity	Alfa	67	67	65	66	59	62
		(100)	(100)	(100)	(100)	(100)	(100)
	Charlie						
Secondary caries	Alfa	67	67	65	66	59	62
		(100)	(100)	(100)	(100)	(100)	(100)
	Charlie						

^s^ Indicates significant difference in comparison with baseline according to Cochran’s Q test fallowed by McNemar's test (p<0.05) FB: Fuji Bulk, GP: G-ænial Posterior. The outcomes were scored as alpha: clinically very good, bravo: clinically good, acceptable, charlie: clinically unacceptable.

Recall rates were 100% for 6-month and 93% for 12-month assessments. At 6-month evaluations, one (1.5%) GP and two (3%) FB restorations lost retention ( *p* >0.05). At 12-month recall, only one FB (1.7%) lost retention. The rates of cumulative retention loss after 12 months were 4.9% for FB and 1.6% for GP ( *p* >0.05).

At 6-month assessments, nine FB (13.8%) and three GP (4.5%) restorations presented bravo scores for marginal adaptation ( *p* >0.05). At 12-month examinations, FB (30.5%) restorations exhibited higher bravo scores than GP (6.5%) restorations for marginal adaptation ( *p* <0.001).

Regarding marginal discoloration, four FB (6.2%) and two GP restorations (3%) showed bravo scores ( *p* >0.05) at 6-month evaluations. After 12 months, FB restorations (20.3%) demonstrated higher bravo scores than GP restorations (6.5%), and the difference was statistically significant ( *p* =0.024).

At 6-month examinations, only two FB restorations showed bravo scores for surface texture. At 12-month assessments, a total of 10 (7 FB, 3 GP) restorations were scored as bravo, and the difference between the two groups was not statistically significant ( *p* >0.05).

All restorations were scored as alpha regarding secondary caries and postoperative sensitivity after 12 months.

McNemar’s test presented significant changes in marginal adaptation and marginal discoloration after 6 and 12 months for FB restorations ( *p* <0.05). GP restorations presented significant changes in marginal adaptation and marginal discoloration after 12 months ( *p* <0.05), compared to baseline.

## Discussion

This clinical trial was the first study to compare this new conventional GIC and a resin composite combined with a universal adhesive in NCCLs of patients with systemic diseases. The null hypothesis was partially accepted. Although the resin composite presented better results to restorative conventional GIC for marginal adaptation and marginal discoloration, no significant differences in retention between the two restorative materials were found.

According to the manufacturer of the new conventional GIC, Fuji Bulk was suitable for patients with systemic diseases for its high resistance to acidic oral environment and high fluoride release capacity. Considering the biocompatibility and chemical adhesion benefits of GICs, these declarations were the main reason to evaluate this product in patients with systemic disease who have a strong requirement for bioactive, biocompatible, maintenance friendly, and long-lasting restorations.

A wide range of restorative materials are available, and clinicians have the opportunity to choose the most suitable treatment for each individual patient. As resin-based materials and amalgam present minor biological side effects, ^26 , 27^ GICs have become popular for the restoration of different type of cavities. ^28^ Their advantages over resin composite restorative materials include fluoride release, chemical adhesion to tooth tissues, and similar physical properties to dentin. Moreover, fluoride release results in an anticariogenic environment that promotes remineralization. ^28^ However, undesirable esthetic properties of GICs are still a problem, hindering their use in patients with high esthetic demands. In this study, all restorations were placed in posterior teeth, as the new conventional GIC did not have an acceptable esthetic appearance.

Ozgunaltay and Onen ^29^ (2002) reported that GIC showed a lower incidence of alpha scores for color match and marginal discoloration than the resin composite. In another clinical trial, resin composite restorations presented better marginal adaptation than GIC. ^30^ In our study, the conventional GIC clearly demonstrated higher bravo rates for marginal adaptation than the tested resin composite. Marginal adaptation failures are mostly due to thermal and mechanical stresses in the oral cavity. Furthermore, water sorption, hydrolysis, and viscoelastic properties of the restorative material affect the marginal adaptation of NCCLs. ^31^ In this study, bravo rates of marginal discoloration were consistent with marginal adaptation rates, and the similar ratings indicated the significance of fine marginal adaptation, leading to marginal discoloration. ^31^ Sidhu ^32^ (2010) investigated clinical outcomes of GICs and reported that they show better retention and lower secondary caries and postoperative sensitivity rates than resin composite restorations. However, marginal adaptation and discoloration, surface properties, and color stability of GICs did not present good results as other criteria in long-term clinical investigations.

Several systemic diseases affect saliva flow and periodontal status of patients. Particularly, chronic diseases such as hypertension ^33^ and diabetes mellitus ^34^ may promote tissues (salivary glands) dysfunction. Drug-induced hyposalivation can be a problem for many types of medications, such as antihypertensive, antidiabetic and psychotherapeutic drugs, and antihistamines. ^35^ After radiotherapy ^36^ or chemotherapy, ^37^ xerostomia may occur as a side effect. Such issues may have an adverse effect on patients’ quality of life and longevity of restorations. In addition to poor quality of life, hyposalivation leads to lower salivary pH, reduced buffering capacity, decreased oral clearance, and reduced immune defense function. Therefore, the type of restoration chosen is crucial for patients with systemic diseases or in remission after cancer treatment. In this study, NCCLs of 16 patients with hypertension, four with cardiac diseases, six with cancer in remission, five with diabetes mellitus, two with rheumatoid arthritis, two with chronic obstructive pulmonary disease, and one with lupus erythematosus were treated. Some patients had more than one of the diseases mentioned. The medications for these diseases may cause dehydration, affecting periodontal response, and resulting in poor oral health. On the other hand, these systemic diseases may also lead to non-plaque-induced gingival inflammation due to altered immune response. ^38^ In this study, the gingival status of patients revealed slight inflammatory symptoms, even one week after prophylaxis, which could be related not only to their insufficient oral hygiene but also to oral sequences of their systemic diseases. This study revealed substantive outcomes for the challenging host conditions which have not been examined previously for the restoration of NCCLs, and this must be considered while interpreting the results.

The adhesion capability of restorative systems is related to dentin hydration, sclerotic dentin formation, and elastic modulus of the restoration materials. ^39^ The dehydration of teeth due to xerostomia may have an adverse effect on the adhesion and integrity of restorative materials. A systematic review ^18^ stated that restoring NCCLs using GIC resulted in better retention outcomes compared to a two- or three-step etch and rinse adhesive system. The studies reported that the elasticity of GICs similar to dentin and their better adhesion to calcified tooth tissues compared to adhesive systems may be the main reasons for the superior results of GICs. ^18^ On the other hand, the more rigid characteristics of resin composite materials and the hybridization of dentin and micromechanical resin tags in enamel are advantages of resin-based materials and adhesive systems. ^40^ Phosphoric acid etching and adhesive systems may lead to marginal adaptation of the restorative resin composite. ^30^ Another clinical trial demonstrated that GIC restorations present higher bravo scores and higher rates of retention loss than resin composite restorations in NCCLs, ^30^ similar to our study after 24 months. However, the examiners concluded that patients with heavy bruxism may explain the higher rate of GICs loss. Conversely, Vaid, Shah and Bilgi ^41^ (2015) reported no significant difference between GIC and resin composite restorations regarding clinical performance after 12 months.

The literature presents few reports of restorative treatment in patients with systemic diseases. ^42 - 44^ In patients with high caries risk, the placement of a viscous GIC is able to prevent the occurrence of secondary caries. ^42^ McComb, et al. ^43^ (2002) conducted a clinical trial in Class V cavities of patients who received radiotherapy and the results showed that retention loss occurred in only one resin-modified GIC restoration and eight resin composite restorations. None of the conventional GIC restorations failed. The resin composite group exhibited a significantly higher rate of retention loss after 24 months. A clinical investigation comparing GIC and amalgam restorations in xerostomic cancer patients reported that patients who did not use topical fluoride as directed, amalgam restorations showed significantly higher retention loss than GICs. ^44^ Although previous studies have also presented positive findings regarding GIC restorations, it is impossible to directly compare these findings with those of our study, considering the diversity of test conditions, cavity types, and restorative materials.

Our patients received oral hygiene training, but 3 of 28 patients developed new caries in their other teeth at 12-month evaluations. Their systemic conditions and the medications were thought to be the reasons for this in addition to their low ability to conduct personal oral hygiene as desired. The use of GIC restorations may contribute to oral health of patients with systemic diseases and cancer patients in remission after chemotherapy and radiotherapy treatment because of their ability to release fluoride and their improved retention rates. ^18^ Furthermore, the time required to place a restoration in these patients is another important factor. The major advantage of GIC used in this study was the short-working time and lack of requirement for etching and adhesive protocols. Restorative treatments must be finished within a short time, when dealing with patients with serious health problems and medications.

Some limitations of this study must be considered when interpreting the results. Firstly, our findings cannot be generalized to healthy patients of varying ages, as we focused on patients with chronic systemic diseases within a limited age range (50–69 years). Another limitation was the difference in systemic diseases of the participants. Although all of the included systemic diseases share the characteristic of reducing the saliva flow rate, ^45 - 49^ their diverse mechanisms and/or medications may have affected the results. Moreover, the assessments of participants’ individual caries risk, particularly their dietary habits, which could also influence the study outcomes, were not examined. The short evaluation period represents another potential limitation of this study. However, limited clinical data on this new conventional GIC are available, and there have been no clinical results published to date. Therefore, further long-term studies addressing specific systemic diseases are required associated with discussions on the differences or similarities of the results.

Although this study yielded promising results for this new conventional GIC for the restoration of NCCLs in patients with systemic diseases at 12 months, further evaluation of its long-term performance is required.

## Conclusions

The resin composite presented superior results to the conventional restorative GIC for marginal adaptation and marginal discoloration. Both materials successfully restored NCCLs in patients with systemic disease during the 12-month evaluation period. Significant changes were observed in marginal adaptation and discoloration for both restorative materials tested over 12 months.
